# Cyber security threats: A never-ending challenge for e-commerce

**DOI:** 10.3389/fpsyg.2022.927398

**Published:** 2022-10-19

**Authors:** Xiang Liu, Sayed Fayaz Ahmad, Muhammad Khalid Anser, Jingying Ke, Muhammad Irshad, Jabbar Ul-Haq, Shujaat Abbas

**Affiliations:** ^1^School of Economics and Management, Fuzhou University of International Studies and Trade, Fuzhou, China; ^2^Department of Engineering Management, Institute of Business Management, Karachi, Pakistan; ^3^Faculty of Business and Management Sciences, Superior University, Lahore, Pakistan; ^4^School of Public Administration, Xi’an University of Architecture and Technology, Xi’an, China; ^5^School of Business, Xiamen Institute of Technology, Xiamen, China; ^6^Department of Management Sciences, University of Gwadar, Gwadar, Pakistan; ^7^Department of Economics, University of Sargodha, Sargodha, Pakistan; ^8^Graduate School of Economics and Management, Ural Federal University, Yekaterinburg, Russia

**Keywords:** cyber security, e-commerce, social engineering, denial of services, malware and attacks on personal data

## Abstract

This study explores the challenge of cyber security threats that e-commerce technology and business are facing. Technology applications for e-commerce are attracting attention from both academia and industry. It has made what was not possible before for the business community and consumers. But it did not come all alone but has brought some challenges, and cyber security challenge is one of them. Cyber security concerns have many forms, but this study focuses on social engineering, denial of services, malware, and attacks on personal data. Firms worldwide spend a lot on addressing cybersecurity issues, which grow each year. However, it seems complicated to overcome the challenge because the attackers continuously search for new vulnerabilities in humans, organizations, and technology. This paper is based on the conceptual analysis of social engineering, denial of services, malware, and attacks on personal data. We argue that implementing modern technology for e-commerce and cybersecurity issues is a never-ending game of cat and mouse. To reduce risks, reliable technology is needed, training of employees and consumer is necessary for using the technology, and a strong policy and regulation is needed at the firm and governmental level.

## Introduction

Technology contributes a lot to our daily life. One of the significant contributions of technology is its applications to the way of doing business (Wang et al., 2022). It has shifted the traditional methods of doing business to the next level. New technologies influence the quality and cost of products and services and business means (Thomson et al., 2022). Business means exchanging something for something; to be more specific, it refers to selling and buying products or services in exchange for money (Burton, 2007). As discussed earlier, the way of doing business has changed due to the application of technology; the business activity involving using or applying electronic technology is known as e-commerce or e-business (Reynolds, 2000). In e-commerce, activities are completed online through the internet. Primarily, e-commerce uses a website, but other technologies such as email, etc., can also be used. Three main parts of e-commerce are the electronic market, online retailing, and online auctions. A customer can buy a product or service distantly by using the application or technology offering the product (Khurana, 2019). E-commerce is still evolving with the development of new technology and its applications and has attracted researchers from various areas like business and technology to enhance the process and make it more beneficial and profitable. But these developments have also brought some challenges to the industry (Jennifer, 2022). One of the challenges is “the cyber security concern” in e-commerce (Mishra et al., 2022) which is one of the most critical and common concerns it faces.

E-commerce business entities and customers are always the targets of cybercriminals and cyber-attacks (D’Adamo et al., 2021). According to a report, 83% of the United States retailers are vulnerable and could easily be attacked (Security Magazine, 2020). Attackers usually attack customers’ private data, which is the most valuable asset in e-commerce. They can either steal the data from the database of online stores, malware, ransomware, and e-skimming. They can also attack in the form of distributed denial of services (DDoS) or Pishing (Bigcommerce, 2022). This is clear that with the advent of business with the help of technology like e-business and e-commerce, opportunities are reaching us more rabidly but not in the absence of issues like cyber security, etc. Like the e-commerce organizations, cybercriminals are also constantly enhancing their technology and skills to find vulnerabilities in the existing system of e-commerce and take advantage of them (Jang-Jaccard and Nepal, 2014). Therefore, this is necessary to explore technology’s pros and cons and address the issues.

It is necessary to highlight here that using advanced technology for addressing the issues of cybersecurity is expensive and most of the e-commerce organizations cannot afford. Many organizations often ignore this control on cybersecurity threats due to its huge costs but they also ignore the returns which may gain the organizations in the longer term (Koomey, 2012). Without a doubt it is true that invest in technology ensures security to al large extant yet it is difficult for smaller and new organization to adopt (Dobrowolska, 2020).

### Problem statement

Even though technology provides tremendous opportunities for the business sector, the challenges accompanying these opportunities cannot be ignored. One of the challenges is in the form of a cyber security threat, the intensity of which is increasing day by day.

### Objectives

The research aims to explore the concerns about cyber security threats in e-commerce with a focus on social engineering, denial of services, Malware, and Attacks on Personal Data and provide a managerial solution.

### Research questions

i. What are the concerns about the cybersecurity threats in e-commerce?
ii. How cybersecurity threats can be addressed and minimized?

This conceptual analysis aims to contribute to understanding cybersecurity in e-commerce. Many of today’s researchers focus on technology’s support in business and ignore the challenges technology is bringing to the company. This work highlights cybersecurity as one of the most critical issues related to technology used in industry (e-commerce). It is focused on some cybersecurity issues, e.g., social engineering, denial of services, malware, and attacks on personal data. Although the scope of cybersecurity is huge, we only discuss some most common types of security breaches. We base this analysis on multiple data sources like books, journal articles, magazines articles, newspapers, blogs, etc. to answer the research questions.

## Theoritical background

### Cyber-attack theory

The cyber-attack theory (CAT) believes that information is the central part of any cyber-attack and states that the success of cyber-attacks depends on the information owned by the attackers at the time of the attack and the information modified or gained during the attack (Zhuang et al., 2015). Each system has configuration information that plays a significant role in a cyber-attack. And it is necessary for a cyber-attacker to have this information. This information includes the information about the system, i.e., configuration information, the system data, etc. CAT describes any system or device to be targeted by the set of information parameters, which the attackers want to gain or modify. Furthermore, the attackers have also information about likewise systems, technical skills, etc. which is helpful in conducting such attacks (Zhuang et al., 2015).

### Information security theory

The *information security theory* (IST) states that “Information security is a conscious or subconscious process in which people and organizations attempt to create sustainably viable resources, from information” (Horne et al., 2016). According to the objectives of information, individuals and organizations protect information from risks and threats by applying suitable control measures. Keeping the information protected according to the need of organization and individual make the information sustainable resources. To be more specific, Information security focuses on the protection of information, suitable for the type and sensitivity of the information and its strategic use for the organization (Horne et al., 2016).

### System theory

The system-theoretic process analysis is an approach that takes the interaction of each component of a system into account to make a system safer and more secure (Thomas, 2016). It is developed by Leveson to find out hazardous states and unsafe control actions which cause accidents or system losses. In addition, it also generates comprehensive safety requirements to stop the happening of known hazardous scenarios (Leveson, 2004). It integrates factors like software, hardware, human, organizational and safety, etc. for the identification of potential threats and risks (Leveson, 2004).

### Causal analysis based on system theory

The causal analysis based on system theory theory states that in order to minimize the risks of accidents and losses, the causes must be identified and analyzed at each component of the system. The objective of this theory is to maximize the learning from incidents and accidents. Although there are some critics of this theory and they believe that it produces too much information to be managed. Yet, it is very helpful in identifying the root cause of any incidents, and the expenditures made in finding those causes or root causes save time and money in the long run (Henderson, 2013).

The above theories show that there are some important factors that must be understood to establish a safe system. For example, the CAT and IST focus on the information and say that in order to make an incident-free system the information must be kept out of the reach of the attackers. Without enough information and knowledge, the attackers are either unable to enter the system or unable to harm a lot. The system theory focuses on the safety measures to be taken at each component of the system, to make the system more secure and protected, and out of the reach of attackers. Even then if an accident occurs, the case analysis theory emphasizes the lesson learned and digging into the root cause of the accident, to plan for the future. Our framework is based upon these theories which is given at the end of discussion section.

## Literature review

### E-commerce

**FIGURE 1:**
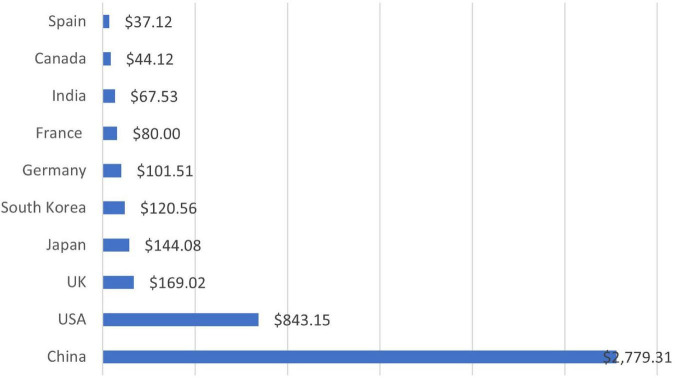
E-commerce sale by country (OBERO, 2022a).

E-commerce is an enormously growing field that came into being due to the advancement and convergence of technology and the internet, where people do many activities related to commerce. In other words, e-commerce refers to the selling and buying of products online. It involves an online money transfer in exchange for completing the business activity. E-commerce uses digital means to develop and perform different actions and transactions among organizations or groups or between a firm and a customer. According to a study, there are more than 12 million – 24 million e-commerce websites across the globe (Gennaro, 2022). Figure 1 shows the country-wise e-commerce sale in 2021 (OBERO, 2022a).

**FIGURE 2:**
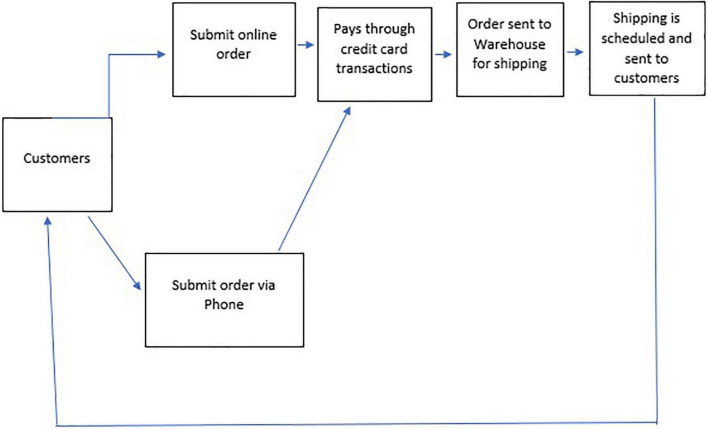
E-commerce workflow diagram (Smart Draw, 2022).

In e-commerce, the business process of buying and selling is completed with the help of the internet. The significant e-commerce activities include a selection of a specific product, money transfer, and data exchange (Ahmadian, 2021). Other activities include marketing through the internet, online management systems, and automatic systems for data collection. E-commerce is helping businesses by enlarging their market scope and size; and reducing operating costs and barriers (Lorette, 2022). The research shows that it positively impacts the economy (Anvari and Norouzi, 2016). In e-commerce, a customer buys directly from the online store using mobile applications and websites. Communication can take place through chatbots, live chat, or voice assistants. The Figure 2 (Smart Draw, 2022) below summarizes the framework of the e-commerce business process from a customer.

**FIGURE 3:**
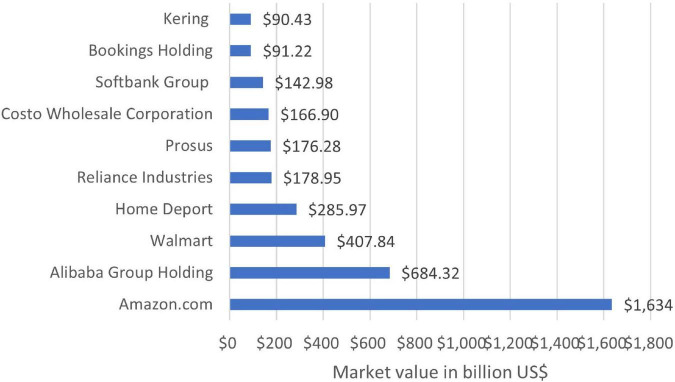
Top e-commerce companies by market value (OBERO, 2022b).

The world is shifting from in-store to online shopping, and big companies like Alibaba, Amazon, etc., are leading the transition. Due to this shift, technological advancements are being made to further online business processes (Hooks et al., 2022). E-commerce provides an ease for customers to buy something and has also proved itself one of the powerful agents for business transformation (Li X. et al., 2022; Thomson et al., 2022). The market value of e-commerce in 2021 is given in the following Figure 3. It shows that Amazon has the largest share, with a value of 1,634 Billion USD (OBERO, 2022b).

Due to the rapid growth, the firms upgraded their networks, operations, etc., to provide better services to suppliers and customers. E-commerce technology made yesterday’s impossible goals for business firms possible by providing them with many opportunities to find and capture new markets and attract customers beyond boundaries (Snihur et al., 2021; Giorgi et al., 2022). Although doing e-commerce has many advantages for business firms and customers, it is impossible without a sophisticated approach to security (Dupont, 2012).

There are four main market sections where e-commerce operates. These sections are Business to Business, where the sale of products is between businesses; Business to Consumer, which involves sales between businesses and consumers; Consumer to Consumer, which allows sale between individuals, and Consumer to Business, where individuals sell to businesses (Shopify, 2022).

**FIGURE 4:**
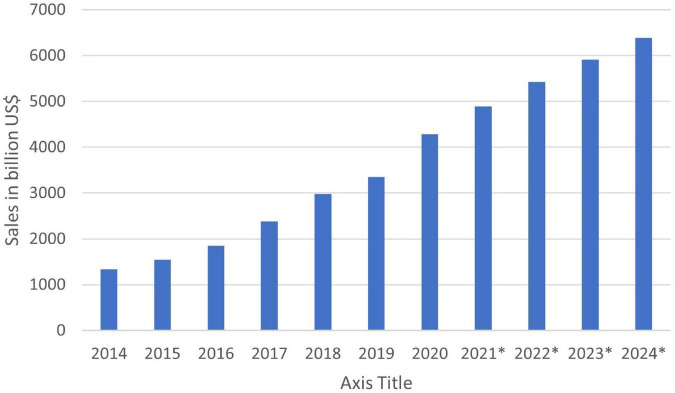
E-commerce sales worldwide (Statista, 2022).

It is important to note that in 2020, the e-commerce sales were 4.28 Trillion USD and are expected to reach 5.4 Trillion USD. The e-commerce share was only about 469.2 billion USD in the United States in 2021. The Figure 4 below shows e-commerce statistics from 2014 to 2024 (Statista, 2022).

The trends and statistics show that e-commerce is a growing field of doing business and is not limited to some specific areas. It is typical for where internet and technology are available across the globe. For example, an industry like tourism is also adopting technology and changing its traditional business. Now sale and purchase of tickets, hotel reservations, etc., can be made with the help of the internet and the relevant technology. The market size of the global online travel agent sector is about 432 Billion USD, the online travel booking platform industry worldwide is about 517 Billion USD and the revenue share of online sales in the global travel and tourism is about 65%.

Therefore, e-commerce technology and firms must be capable of doing business without difficulty and provide their customer with the best possible experience. But as said earlier, as technology is involved between the purchasers and buyers, the activity completes remotely after sharing the required information. E-commerce invites many threats, and cyber security is the most common and severe in them.

### Cyber security

One of the most significant challenges e-commerce faces from the beginning is cyber security threats (Kianpour et al., 2021). Cyber security protects computer systems from information disclosure, misdirection, damage, or theft of electronic data, software, or hardware (Schatz et al., 2017). In e-commerce, it is all about electronic security related to e-commerce activity. Business firms continuously invest in technologies to prevent cyber threats, but cyber actors obtain access to business systems and data. The landscape of cybersecurity issues is evolving as cyber actors are searching for new vulnerabilities through different means. On one hand, malicious actors are enhancing their skills and on the other, they are adopting advanced technologies and techniques to target various organizations (Wirth, 2017). Almost all organizations using internet or computer connectivity, including healthcare, financial firms, transportation, government, and manufacturing industries, are targeted continuously (Strategic Technologies Program, 2022). During the Covid-19 pandemic, the number of attacks were increased by 600% due to the increase in the number of users and dependency on technology. The cost of cybercrime was 3 Trillion USD in 2015, estimated to be 6 trillion USD in 2021 (Morgan, 2017). It is estimated that by 2025, the cost will be 10.5 Billion USD for businesses which is more than the economy of any country after the United States and China (Expert, 2021). This shows how cybersecurity is essential in the current digital and technological era for businesses and organizations, especially those involved in e-commerce (Team, 2022).

It is essential to understand why cybersecurity breaches occur. There are three main reasons for cybersecurity-related issues;

Humans are listed as a significant source of CS by the United States and the United Kingdom (Dykstra, 2017). According to a study, humans are more vulnerable to cause a security breach than technology, i.e., 86, and 63%, respectively, (Metalidou et al., 2014). Another study shows that 80% of cyber-attacks occur due to human-enabled errors (Saeed et al., 2013). Human technology interactions invite security risks, and firms continuously struggle to prevent and mitigate human behavioral-based threats to information security (Nobles, 2015). To obtain a competitive advantage and capture a significant share in the market, business firms adopt and invest in advanced information systems, which often leads to an increase in human mistakes when using the technology. Customers and employees are the weakest link in risk and security management (Alavi et al., 2016). With each passing day, the CS threats are increasing, and firms are continuously adopting and leveraging new technologies to prevent them (Neely, 2017). In addition to inducing the latest technologies to counter the threats, it is also necessary to minimize the behavioral risk associated with humans by adequately training and enhancing their understanding of their interactions with the organization’s information systems (Metalidou et al., 2014). As much as human factors are involved in the concerns related to cybersecurity, most organizations have failed to invest in humans to address the issue (Alavi et al., 2016). It is clear that humans cause cybersecurity threats, as a customer may share their information or data incorrectly, with the wrong person, or to a vulnerable information system. Humans as an employee of the business organization may not be able to use the technology properly and may invite severe cybersecurity threats for both the organization and customer. Last but not the least, the employee may use the information (consumer and organizational) for their personal gain. In all form, human poses a serious threat and it is a big challenge for e-commerce to address.

*Technology:* In addition to the human factor associated with cyber threats, the second potential threat is the technology itself. Cybercriminals are taking advantage of vulnerabilities induced by the technology, hyper-connected systems, human-enabled errors, and organizations not prepared to prevent or counter such attacks. The most common cyber threats noted in 2021 are phishing, social engineering, credential theft, and compromised or stolen devices with 57, 30, and 33%, respectively, (Team, 2022). Other common threats are spyware, ransomware, trojans, etc. (kaspersky, 2022). A study shows that “-about 81% of breaches resulted from weak or stolen passwords, 62% of breaches stemmed from hacking, 51% of breaches involved malware, and 43% of breaches were social engineering attacks” (Verizon, 2017). One of the most significant sources of cybersecurity issues is the hyper-connectivity of technology in the modern era and the dependency of business and commerce on these hyper-connected systems (Abdel Hakeem et al., 2022). It refers to the networked societies or technologies with each other and the various ways of communication like email, instant messaging, etc. In the context of e-business or e-commerce, it is about the connectivity of an organization’s information system having enormous records with the outside world. This connectivity makes it vulnerable to cybercriminals who take advantage of it.

*Non-preparedness:* Another reason for cybersecurity threats is non-preparedness (Pusey and Sadera, 2011). Many organizations are unprepared for cyberattacks (Abdelhamid et al., 2019). Either they lack advanced protocols and tools to prevent counter-cyberattacks (Zwilling et al., 2022), or do not respond well (Akpan et al., 2022). Due to their un-preparedness, the attackers take advantage of the opportunity.

**FIGURE 5:**
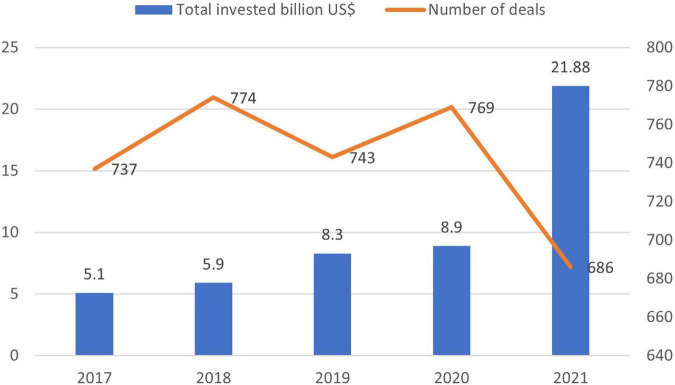
Investment in cybersecurity (Metinko, 2022).

The severity of non-preparedness in cybersecurity threats in the modern electronic era is evident from the statistics that only in 2021, 21.8 Billion USD was poured into cybersecurity compared to 5.1 Billion USD in 2017, 5.9 Billion USD in 2018, 8.3 Billion USD in 2019, and 8.9 Billion USD in 2020. In addition, if we look at the data, the last quarter of 2021 witnessed the highest amount of 7.8 Billion USD investment in cybersecurity as shown in Figure 5 (Metinko, 2022).

**FIGURE 6:**
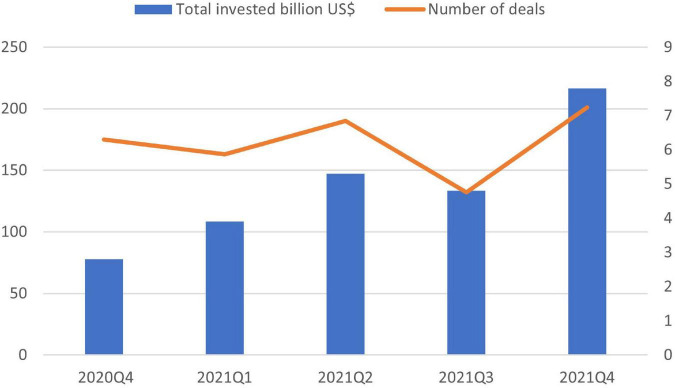
Investment in cybersecurity quarterluy (Metinko, 2022).

Figure 5 above shows that the investment in cybersecurity is increasing yearly with rapid growth in 2021. It is also evident from the Figure 6 that in each coming quarter, the amount for cybersecurity funding increased with a sudden high increase in the last quarter of 2021 from 3.9 Billion USD in the first quarter, 5.3 Billion USD in the second quarter, 4.8 Billion USD in the third quarter and 7.8 Billion USD in the fourth one (Metinko, 2022). To summarize the discussion, the challenge of the cybersecurity landscape is getting worse, and the actors are becoming more experienced and acquiring more sophisticated ways for attacks. This not only increased the number of data breaches, etc. but also threatened the e-commerce organization.

#### Social engineering

Social Engineering is the most common type of scam, that cybercriminals use (Nick Galov, 2022). It is any activity that influences a person’s behavior for taking an action that is not necessarily in their interest (Social-engineer, 2022). It is the psychological manipulation of compelling customers to perform various tasks, activities, etc., and to expose or reveal their confidential information. It may be one step trick or maybe of many steps, but the purpose remains the same, to collect conditional details or to get access to the system (Anderson, 2008). The simplest example of social engineering is the “forget password” option which, after clicking, may direct the user to a malicious link and grant access to the attackers to the user account or system.

Similarly, the original user will no more able to access the invoice (Purplesec, 2022). The target of social engineering may be a firm’s top executives or a student. In other words, anyone can be a target of social engineering attackers (Sanders, 2022). The severity of social engineering can be seen from the statistics that about 98% of cyber-attacks come from this threat (Purplesec, 2022). The most common techniques used in malicious social engineering are:

*Phishing:* Where the attacker sends emails showing that it is coming from a reputable source and ask for information. Through this process, the criminal gathers personal data and uses it accordingly (Phishing, 2022). Phishing websites are 75 times more than malware, and about 70% of the companies worldwide were a victim in 2020 (Galov, 2022). Only in 2020, business losses were $1.8 billion due to it (Purplesec, 2022).

*Vishing:* Where the attacker uses a telephone call to attempt or to encourage an action. The purpose is to gather data and obtain valuable information necessary for a firm or individual to compromise (Olson, 2018). Only in 2021, there was a 554% increase in the volume of vishing attacks, which is about 27% of the overall response-based threats. It can be seen from these statistics that it will further increase in the future (LaCour, 2022).

*Impersonalization:* The attacker presents itself as another person or firm and gets socialized to obtain and gather information or access to a firm, system, etc. (Easydmarc, 2021). Between 2020 and 202, there was a 131% increase in personalization, costing 1.8 Billion USD to the targeted enterprises (Securitymagazine, 2022).

*Smishing:* Where the attacker is sending messages on the phone to influence the immediate actions of a victim, like direction to visit a malicious website, downloading something, etc. (Hughes, 2021). It is reported that smishing scams only rose by about 328% in 2020.

In e-commerce, when a customer enters a website or page, the attackers socialize and get the data after making a trustful relationship and then use the information or data for personal use. It is not necessarily that the victim must be a new one, but maybe anyone, regardless of his experience, education, and position, might be on target.

#### Attack on customer personal data

Targeting personal data is also one of the significant challenges e-commerce is facing. As the world is getting more digitized, the amount of data (firm’s data and customer data) shared, stored, and saved on systems and online; is also increasing daily to a tremendous huge volume (Zende, 2022). Similarly, access and utilization of the data and network are also growing. This increases the risk of cybercrime in the form of an attack for retrieving confidential data and also decreases the trust of customers and firms in each other (Hussien et al., 2022). In e-commerce, the customer must share their private information with the organization, making it capable of knowing and recording much information about the customers (Vasupula et al., 2022). For example, home address, phone number, bank card number, date of birth, etc. The online store or company may also record your purchasing history and compare it with your buying details.

There are two main types of attacks on personal data:

1. The online store or organization can use the customer personal information without consent.
2. The data can be attacked and used by cyber attackers, who do not belong to the online firm but from outside and want to steal data.

Only in 2019, around 15 billion data records were compromised (Security Magazine, 2020). It means that concern regarding the attacks on customer personal data is the number one challenge for e-commerce. The customers and e-commerce companies need to understand the risks associated with customer data and the cost of the breach (Varga, 2021). It is a fact that we are living in an information age and information is the most precious asset of this era. Based on the information, organizations design their strategies, plan their products and services, and invest. Also, sharing information with someone or some organization needs a significant trust from the party sharing its information and doing so for a purpose. It is the organization’s responsibility to keep the information safe and protected from illegal use to maintain the customer’s trust and use it in a competitive strategic manner. Suppose the attackers successfully steal the customer information. In that case, it will hurt the customer’s trust and the organization will be no more able to behave in a competitive strategic manner in the market.

#### Distributed denial of service attacks

It is a type of cyber-attack in which the criminal tries to make the service or system unavailable to the users by disrupting the services through different means (Ncsc, 2022). The most common denial of the service attack method is sending a flood of requests to overload the system and prevent legitimate submissions. Most traffic flooding comes from more than one source, and it is difficult to stop the attack (Fortinet, 2022). In a DDoS attack, the attackers continuously send requests from many authorities to get the web resource down. In e-commerce, for example, they flood the online store, etc., with massive traffic and make the customers unable to purchase something (Anshari et al., 2022). This leads to the disability of the online firm for hours or even for several days. And if the attack is in peak season, it is more annoying and severe; it may cost a considerable amount in the form of customer and income loss (Dahiya and Gupta, 2020).

The primary purpose is to make service delivery impossible by thwarting online firm or store access. It can be of many forms and depends upon the purpose of the attackers and the nature of the e-commerce firm or store (Mishra et al., 2022). There are three main types of DDoS attacks (Fruhlinger, 2022):

1. *Volume-based:* The attackers use considerable traffic to make a resource (server or a website) unavailable (Fruhlinger, 2022).
2. *Network layer:* The attackers use many data packets to target the network infrastructures (Gargar, 2021).
3. *Application layer:* Here, the attackers use maliciously crafted requests to flood the applications and make them unavailable to genuine customers (Gargar, 2021).

It is one of the most significant cybersecurity issues of e-commerce, where the criminals make the e-commerce source or resource unavailable or unreachable to the customers. Availability of service is an essential part of attracting users. For example, if a service is available and its quality is better than that of competitors, people will get automatically drawn to it. In other words, if a service is unavailable, it will not attract people and if the quality is not good, again, the people will opt for a better service. In both cases, there is a loss. Therefore, e-commerce organizations must make sure that their service is available with better quality than competitors. Their system must have the capability to detect DDoS attacks and responds promptly.

#### Malware (malicious software)

Malware is any software that could infect computers, and cybercriminals use it to insert it on target websites (CYBER EDU, 2021). The primary purpose is to obtain personal data like passwords, account details, money stealing, or blocking the system owner from using it (Lutkevich, 2021). Usually, this way, the user gets misled and is redirected to another website or page. Malware attacks are widespread attacks that execute illegal activities on the victim’s system. It may be ransomware, control of the device, or spyware (Rapid7, 2022). Malware is designed to interrupt or malfunction a server, computer, or computer network. After gaining unauthorized access to the system, it breaks security and privacy and obtains private information (Brewer, 2016). Common types of malware are worms, viruses, trojans, ransomware, horses, spyware, rogue software, scareware, adware, etc. It is challenging to address all types of threats with the same strategy because each type needs its defense strategy like antivirus, firewalls, algorithms, etc. (Xiao et al., 2020). It is a severe problem for e-commerce (Kim et al., 2018). The number of attacks through malware is a serious threat to e-commerce as the number of attacks is increasing yearly by a significant proportion. There were 670,000,000 malware variants in 2017, almost double the number in 2016 (Xiao et al., 2020).

The development and application of modern technology have a lot of advantages for the business sectors but also bring threats in the form of malware. The number of incidents and threats increases yearly as the technology develops and its applications enter new boundaries. It is necessary for the e-commerce organizations that the technology they are using must have the capabilities to detect malicious software and prevent them from entering their system. Again, it can target the organization and its customers; both need to know about it and use secure technology and service.

## Discussion

In the contemporary era, technology is everywhere, in education (Ahmad et al., 2021), assisting in academics and administration tasks (Ahmad et al., 2022) to business (Ibrahim et al., 2014), from marketing to industry (Sayed et al., 2020), from health to space sciences, etc. Trade and commerce are tremendously influenced by digital technologies, which changed the business mood from traditional/conventional to electronic (den Hond and Moser, 2022). Due to technology, not only did the business sector find new opportunities but also expanded beyond geographical limits. Technology enabled the sustainability of e-commerce in the recent Covid-19 pandemic, and enormous growth was witnessed (Stalmachova et al., 2021). But some concerns need to be explored for sustainable and successful e-commerce using technology. The most common among them is cybersecurity threats. E-commerce sites are always targeted for cybercrimes in the form of cyberattacks. Cybercriminals or attackers target e-commerce firms through different means for different purposes. They aim to steal private data like personal information, account details, or financial data and compromise the system not to work correctly. Usually, e-commerce firms of all sizes are on target. The most common cyberattacks are Pishing, denial of service, social engineering, malware, direct access attacks, reverse engineering, spoofing, etc.

The word of e-commerce stands for electronic commerce or the commerce done with the help of electronic technology, the application of technology is increasing daily in e-commerce (Rahman, 2014). Firms are also adopting/implementing technologies without any delay to reach customers and capture market share (Kramer, 2022). There is never-ending competition among the firms in almost all sectors (Wall, 2022). Now each product is available online, from books to medicine, from ticket booking to hotel booking, etc. Also, the customers are looking for their comforts and need fulfillment from buying through trustworthy means (Joyce, 2022). E-commerce is the platform that provides products and services according to the customer’s needs. In an e-commerce environment, the customer can explore the market with the help of a few clicks and quickly find out the difference in product quality, price, and delivery time and compare with the other service providers in the market. So this makes a win-win situation for the customers. But searching different websites/pages and clicking various links are not free from the threats. Sometimes it becomes difficult to differentiate between the actual website and the one aimed at cybercrimes. Sharing information like name, address, account details, phone numbers, etc., may reach the wrong place or person through these websites. Not only the criminals who steal customer information through social engineering, phishing, malware, etc. the same can also attack the e-commerce organization in the same manner (Li and Liu, 2021). Competitors may also pose a severe cybersecurity threat by hacking access to an e-commerce website, DDoS, etc. They may also attack to stole customer information and the selling records of an organization. Such records are the backbone of strategic planning and mean a lot to the e-commerce organization (Hepfer and Powell, 2020).

To address the issue of cybersecurity threats, e-commerce organizations are investing a lot to get rid of it (Team, 2022). The statistics show that the investment to address the issue is increasing each year, but the number of attacks is also growing. It means that the problem can’t be resolved without advanced technology. One reason for this is that new people are getting inclined to avail e-commerce and are more vulnerable than the old user. Hackers, attackers, or cybercriminals are also enhancing their skills and searching for vulnerabilities in the technology, etc. It is a fact that there are many other cybersecurity threats besides social Engineering, denial of services, malware and attacks on personal data were discussed in this study. Based on the review, it is evident that cyber-attacks negatively impact businesses in two ways.

1. The cost of a data breach.
2. Losing the customer trust.

As discussed earlier, e-commerce business firms are continuously investing to address cybersecurity concerns, which are increasing yearly (Vinoth et al., 2022). The governments are also making laws and policies regarding this issue (Luo and Choi, 2022). Still, criminals are also finding new methods to target a customer or firm as technology develops. Similarly, firms support implementing innovative and trustful technologies and tools on their websites to remain competitive and maintain the trust of their customers (Gull et al., 2022). On the other hand, researchers are also continuously enhancing the technology, e.g., work has been done to improve the effect of false alarm detection and then more accurately identify real alarms (Li S. et al., 2022), and various tools were proposed for phishing detection (Gupta et al., 2021). Also, researchers are developing new methods and frameworks to find out the vulnerabilities (Cvitić et al., 2021). Work has been done to develop a botnet defense system to exterminate malicious botnets and make the technology usage more secure (Pan et al., 2021). But on the other hand, the attackers are searching for vulnerabilities, and the never-ending game of mouse and cat continues. Block chain technology is another option for e-commerce trust and security (Centobelli et al., 2021a) and digitalization (Cerchione et al., 2022). It may also shape the future of decentralized technologies also (Centobelli et al., 2021b).

**FIGURE 7:**
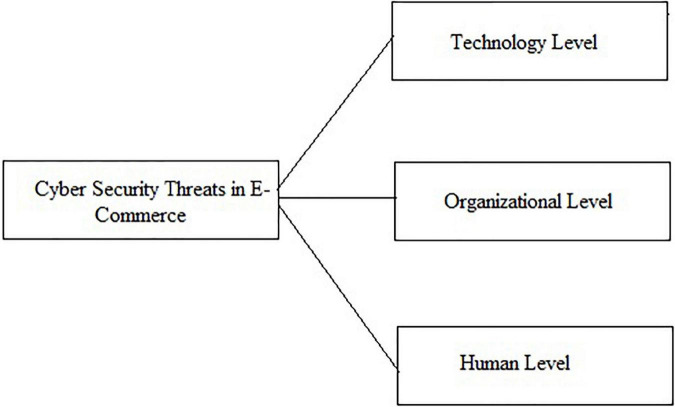
Conceptual framework for cybersecurity in e-commerce.

We divide the cyber security concerns in e-commerce at three different levels and proposed the following framework as shown in Figure 7.

1. *Human:* Cyber security issues occur due to humans (employees, attackers, and consumers) either lacking the proper knowledge and skills to use the e-commerce technology or not following the protocols related to Cyber security (Zhuang et al., 2015). And if they are attackers, then they know more about the technology, organization, and the users of the technology, i.e., they possess more information. Employees and customers, who are using a particular e-commerce technology must have sufficient knowledge, skills, and information to use the technology properly and to complete a business transaction successfully (Al-Ghamdi, 2021). They also need to have information about the technology and organization and must know the vulnerabilities in both. With the help of information, cybersecurity threats could be minimized as the employees and customers will always be alert where there is some vulnerability. And to a greater extent, they are well aware of the attackers, and what and how they attempt (Zwilling et al., 2022).
2. *Organization:* At the organizational level, security concerns occur due to inadequate rules, regulations, and policies to implement the security protocols and use the systems according to the law. If cybersecurity is not the theme of an e-commerce organization’s strategy, it is impossible to address it. Organizations need to invest in training, enhancing security controls and measures, and must continuously be searched for the vulnerabilities and their possible solution at the management level (Roumani et al., 2015). If it ignores the need for something that should be done to address cybersecurity threats, they may become a cause of potential damage in the future (Guembe et al., 2022). Organizations should adopt new procedures and policies to overcome the cybersecurity threats as per market demand, organizational need, and attackers’ skills and knowledge.
3. *Technology:* E-commerce organization often does not invest much to implement a suitable and safe technology, due to which cybersecurity risks increase (Guembe et al., 2022). It is necessary for e-commerce organizations to invest significantly in hiring new and more secured technology (Dobrowolska, 2020). Maybe it is expensive but more beneficial in the longer term (Koomey, 2012).

## Conclusion

The name of e-commerce is attractive and the need of the modern-day business market, but it is facing the challenge of cyber security threats. Although firms continuously invest a lot to address the issue, it is not easy. Personal and organizational data are often the target of cyber-attacks. Without a doubt, technology offers new ways of doing business and provides many additional benefits, but cyber security concerns will always be there. Investing and enhancing the security of e-commerce is substantially essential for getting a competitive advantage and for the success of e-commerce business (Hepfer, 2021). No one can afford the price of customers’ trust; they lose because of the exposition of their data. Strong monitoring protocols must be followed before any mishap on both organizational and customer ends. For example, strong passwords and being cautious about clicking and downloading something. Taking advance precautions and investing in a secure version of the technology in e-commerce is the need of the day.

We conclude that no matter how much the employees and consumers are trained and skilled to do e-commerce, how much the e-commerce firm implements and focuses on the implementation of cyber security protocols and policies; and how much-advanced technology is used for conducting the e-commerce business activities; the challenge of cyber security threats will always be there like a sword to hurt the business and no one knows when.

### Recommendation

With each passing day, the involvement of technology is increasing with a surprising speed in doing business, i.e., in e-commerce. And to be honest, we cannot escape from its applications or ignore its benefits. But the technological transformation in the form of e-commerce has the foremost challenge of cyber security threats. No matter in e-commerce, the technology we are using today, no matter how trained we are to use a particular technology, and no matter what precaution measures we take, the cybersecurity concerns will always be there for various reasons. We put the following four questions before e-commerce organizations to be answered for sustainable and less risky business activities.

1. Is your organization always ahead and aware of cybersecurity concerns and of advanced practices to address them?
2. Do the technology you are using or implementing for doing an e-commerce business enough secured to face cyber-attacks?
3. What was the impact of any cyber-attack on this technology when it was targeted somewhere or in this organization?
4. Do you have an effective and efficient policy or protocols regarding using technology or doing activities to minimize or overcome cyber threats, etc.?
5. Are you prepared for starting or continuing e-commerce without cyber security threats? Are you ready to handle such situations?

### Implication/contribution

#### Theoretical contribution

This conceptual analysis analyzes the cybersecurity threats in e-commerce. It explores that cybersecurity is a potential threat to e-commerce and must attract more attention than the present, according to the statistics analyzed (Furner, 2004).

#### Managerial implications/contribution

The study has the following implications for managers.

1. Without a doubt, modern technology is the need of the day and its applications in business is an irrevocable fact. Managers should take full advantage of modern technology and implement it to capture a larger market and business expansion volume. But they should also be aware of the cyber security threats coming with using and implementing new technology. They should select the appropriate technology to ensure cyber security and train their emplyees how to use it and respond in an unwanted situation.
2. The challenges come with technology, and often, the organization’s employees have less understanding of the new technology. This study highlights the importance of employees’ knowledge about technology usage and managers should provide proper training to the employees to minimize the risks coming from cybersecurity threats.
3. In short, managers can use this work to choose a secured technology for their e-commerce operations and continuously invest in addressing emerging cybersecurity threats.

### Future work

1. Many other factors related to cyber security were not studied in this study due to its limited scope, and they can be explored in future studies.
2. Addressing the questions as recommended above are also significant areas for future work.
3. Quantitative analysis of this study to make it more generalized.
4. Research on block chain technology in e-commerce can be done in future to make it more trustworthy (Centobelli et al., 2021a).
5. Similar research can be done on knowledge management in e-commerce to address the contemporary challenges (Castagna et al., 2020).
6. Research can also be done on the social, economic and environmental issues and impact of cybersecurity and e-commerce.

### Limitation

The scope of cyber security and e-commerce is comprehensive, and this work is limited to its scope as a perspective work. Explorative, qualitative, and quantitative research with a much broader scope is needed to discover other sides of this study.

## Author contributions

SFA and SA contributed to conceptualization, writing – original draft, and methodology. XL contributed to supervision. JK contributed to formal analysis. MA contributed to variable construction. MI contributed to funding acquisition. MI and JU-H contributed to data handling. All authors have read and agreed to the published version of the manuscript.
